# Dynamic and unpredictable changes in mutant allele fractions of BRAF and NRAS during visceral progression of cutaneous malignant melanoma

**DOI:** 10.1186/s12885-019-5990-9

**Published:** 2019-08-07

**Authors:** V. Doma, S. Kárpáthy, E. Rásó, T. Barbai, J. Tímár

**Affiliations:** 1 2nd Department of Pathology, Semmelweis University, 93. Üllői, Budapest, H-1091 Hungary; 2 Department of Dermatology, Semmelweis University, Budapest, Hungary

**Keywords:** Mutant allele fraction, BRAF, Melanoma, Metastasis

## Abstract

**Background:**

Data indicate that primary cutaneous melanomas are characterized by clonal heterogeneity associated with oncogenic drivers. Less data are available on the clonal changes occurring during melanoma progression. We therefore wished to analyse these changes in skin melanomas in common sites of visceral metastases as compared to the primary tumor.

**Methods:**

An autopsy cohort of 50 patients with BRAF- and NRAS-mutant cutaneous metastatic melanomas including 139 visceral metastases was analysed for mutant allele fractions (MAF), determined by pyrosequencing and corrected for tumor/normal ratio. MAF levels were also classified as high (> 40%), medium (15–40%) or low (< 15%).

**Results:**

Contrary to NRAS mutant cases, in BRAF-mutant melanomas MAFs were found to be significantly increased in visceral metastases compared to the primary due to the significantly higher levels in lung-, adrenal gland-, intestinal- and kidney metastases. The incidence of the three MAF variants in BRAF-mutant primaries was similar, whereas the high MAF cases were found to be increased in metastases. On the other hand, medium MAF levels were more common in case of NRAS-mutant tumors. Only 31.3% of BRAF mutant- and 50% of NRAS mutant cases maintained the MAF profile of the primary in metastasis. In the majority of multiple metastatic tumors, (BRAF:71.8%, NRAS:75%) metastases were relatively homogeneous regarding MAF. However, in 6/32(18.7%) of BRAF mutant cases low MAF primaries switched to high MAF in metastases. In heterogeneous BRAF mutant metastatic cases low to high or high to low MAF conversions occurred in a further 4/32(12.5%) cases in individual metastases as compared to the primary tumors. At lower frequency, in NRAS mutant tumor such changes also observed (2/12,16.7%).

**Conclusion:**

We provided evidence for the selection of BRAF-mutant melanoma cells during metastatic progression to the lung, intestine, adrenal gland and kidney. Our findings suggest that in visceral metastases of malignant melanoma BRAF- or NRAS-MAFs are rather heterogeneous and cannot be predicted from data of the primary tumor. These data may have clinical significance when using targeted therapies.

**Electronic supplementary material:**

The online version of this article (10.1186/s12885-019-5990-9) contains supplementary material, which is available to authorized users.

## Background

It is now evident that solid tumors are genetically and clonally heterogeneous and this heterogeneity is unstable during malignant progression [1]. As a consequence, the genetic portrait obtained from the primary tumor (frequently removed by surgery) may not represent the evolving metastases correctly. In the majority of cases UV-induced human skin melanoma is characterized by founder somatic mutations of two oncogenes, BRAF and NRAS. As oncogenic drivers, one would expect that the primary tumors are dominated by tumor cell clones carrying the activating mutation, however, there are data indicating that this is not necessarily the case; heterogeneity both for mutant BRAF [2, 3] and NRAS [4] may occur in primary melanomas. Detection of oncogenic mutation in the primary tumor is a routine diagnostic procedure in case of melanoma, the result of which is an important issue in treatment planning [5]. However, this can only be justified if the metastases represent the primary tumor accurately. Several studies have compared the primary melanoma to the distant metastases with respect to BRAF and NRAS mutation statuses. Such studies have presented quite controversial data, with some reporting high concordance rate of founder mutations [6], while others have demonstrated significant discrepancies [7–10]. One plausible explanation for these controversial results appears to be the use of molecular techniques which show different levels of sensitivity [5, 11]. However, another feasible explanation could be the changes in the clonal proportion of driver genes of the progressing melanoma to distant metastases [12, 13].

In the past years, several attempts have been made to determine the mutant allele fraction (MAF) of driver oncogenes of melanoma in both primary tumors and metastases [3, 4, 14]. The studied metastases were frequently lymphatic or cutaneous (because of their good accessibility), developing at different time points during disease progression. However, data on the driver oncogene’s MAF of the visceral metastases of malignant melanoma are scanty. MAF of driver oncogenes can be important from the viewpoint of targeted therapies. However, data in the literature regarding melanoma in this respect are also quite rare and controversial [14, 15]. This could be explained by the fact that during targeted therapy of the metastatic disease, MAF data of the primary tumor were used [16] furthermore, the various MAF levels of the treated tumors were not analysed in detail [17].

Oncogene mutations in sporadic tumors can be heterozygous by default, resulting in a mutant allele fraction (MAF) value of 50% in tumors where all tumor cells carry the founder mutation corresponding to (mono)clonality [3, 4]. The MAF value can be higher due to the absolute or relative increase in copy number of the mutant oncogene, although this in itself does not necessarily affect driver clonality. On the other hand, less than 50% of MAFs can be due to subclonality of the driver when the tumor is a mixture of mutant and wild type tumor cells or when the heterozygosity is lost due to loss of the wild type gene copy [3, 4].

In the present study, we used an autopsy cohort consisting of 50 visceral metastatic cutaneous melanoma cases, in most of which multiple metastatic organs were available for driver oncogene’s MAF analysis. Using this cohort we were able to compare the metastases to the primary tumor and the various organ metastases to each other.

## Methods

### Patient selection

Our study was carried out in strict accordance with the Declarations of Helsinki and was approved by the Semmelweis University Regional and Institutional Committee of Science and Research Ethics (IRB, SE TUKEB 114/2012).

Cases were enrolled from pathological FFPE archives of the primary tumors and metastases of autopsy cases from the following institutes [1]: Semmelweis University, Budapest [2], Saint George Teaching Hospital of Fejér County, Székesfehérvár [3], Hospital of Zala County, Zalaegerszeg. The set of matched primary and metastatic tumors consisted of 187 FFPE and two aspiration cytology samples. Patient and sample characteristics are shown in Tables 1 and 2.

**Table 1:** Summary of clinical and pathologic characteristics of the 50 primary melanoma

Breslow thickness (mm), range, SD	4,71 (0,25-24,00)(±3,98)
≤1,00	4 (8)
1,00-2,00	7 (14)
2,01–4,00	18 (36)
> 4,00	21 (42)
Histological subtype	
SSM	20 (40)
NM	20 (40)
ALM	1 (2)
LMM	1 (2)
Unclassified	8 (16)
Anatomic distribution	
Trunk	21 (42)
Head and neck	9 (18)
Extremities	20 (40)
Stage at diagnosis	
IB	4 (8)
IIA	10 (20)
IIB	12 (24)
IIC	12 (24)
IIIA	3 (6)
IIIB	5 (10)
IV	4 (8)
Specific histopathological restrictions	
ulceration	24 (48)
regression	4 (8)
solar elastosis	8 (16)
association with a coexistent naevus	4 (8)
Gender	
Male	34 (68)
Female	16 (32)
Age at surgery (years), range, SD	53 (22–81) (±16,14)
< 50	20 (40)
≥ 50	30 (60)
DNA concentration (ng/ul), SD	134,61 (±115,54)
OS (month), range, SD	45 (1–144) (±35,64)
Tumor content (%), SD	79,1 (±20,14)

Abbreviations: *ALM* acrolentiginous melanoma, *LMM* lentigo maligna melanoma, *NM* nodular melanoma, *OS* overall survival, *SD* standard deviation, *SSM* superficial spreading melanoma

**Table 2:** Characterisation of the metastasis cohort

Distant haematogenous metastases	*n* = 139 (100%)
Main visceral organs:	78 (56)
CNS	38 (27)
Lung	23 (17)
Liver	17 (12)
Other organs:	61 (44)
Adrenal gland	10 (7)
Intestinal tract	8 (6)
Distant skin	8 (6)
Kidney	6 (4)
Heart	5 (4)
Spleen	5 (4)
Pancreas	4 (3)
Bone marrow	4 (3)
Mesenterium	3 (2)
Thyroid gland	3 (2)
Bladder, Submandibular gland, Tongue, Prostate, Caval vein thrombus	1 (1)
DNA concentration (ng/μl), SD	209,43 (±197,47)
Tumor/normal ratio (%), SD	78,8 (±21)

Abbreviations: CNS, central nervous system; SD, standard deviation

### DNA extraction

Before DNA extraction, a section stained with H&E was prepared to label and macrodissect the optimal tumor area and to evaluate tumor/normal cell ratio under light microscope. Tumor- to normal cell ratio was determined by counting nuclei at three 40x lens fields by an experienced pathologist (JT). The mean T/N ratio of this cohort was ~ 80% (Tables 1 and 2). High Pure PCR Template Preparation Kit (Roche Holding Ltd., Basel, Switzerland) was used to isolate DNA, which was quantified using NanoDrop ND-1000 UV-Vis Spectrophotometer (NanoDrop Technologies, Wilmington, DE).

#### RFLP of BRAF exon 15 PCR products

PCR amplification of exon 15 with BRAF specific primers yielded a 197 base pair product. This was analysed using restriction fragment length polymorphism (RFLP) by digestion with TspRI enzyme (New England Biolabs, Ipswich, MA) in order to screen for codon 600 mutant BRAF. Products were separated using 3% agarose gelelectrophoresis, stained by ethidium bromide and fragments were identified based on the estimated length of the separated products. The basis of the method is that V600 mutation abolishes the restriction site resulting in a prominent band of 212 bp of the mutant allele, whereas the wild type of BRAF is completely digested enzymatically, yielding DNA fragments at 125 bp*.*

### Sanger sequencing

The primers were designed for BRAF, NRAS and c-KIT with Array Designer software (Premier Biosoft International, Palo Alto, CA) and purchased from Integrated DNA Technologies (Coralville, IA). Primer sequences were as follows: BRAF exon 15 sense: 5′-TTCCTTTACTTACTACACCTCAGA-3′, BRAF exon 15 antisense: 5′-TGGAAAAAT-AGCCTCAATTC-3′, NRAS exon 2 sense: 5′-TTGCTGGTGTGAAATGACTGAG-3′, NRAS exon 2 antisense: 5′-ATATGGGTAAAGATGATCCGACAAG-3′, NRAS exon 3 sense: 5′-AAACAAGTGGTTATAGATGGTGAAAC-3′, NRAS exon 3 antisense: 5′-GTAGAGGTTAATATCCGCAAATGAC-3′, c-KIT exon 11 sense: 5′- CAGAGTGCTC-TAATGACTGAGAC-3′, c-KIT exon 11 antisense: 5′-AAGCCACTGGAGTTCCTTA-AAG-3′, c-KIT exon 13 sense: 5′-CTTGACATCAGTTTGCCAGTTG-3′, c-KIT exon 13 antisense: 5′-TCCAAGCAGTTTATAATCTAGCATTG-3′. We used primers in 1 μM final concentrations per reaction. Applied Biosystems AmpliTaq Gold 360 Master Mix was purchased from Life Technologies Corporation (Carlsbad, CA). Each reaction (25 μl in volume) contained a minimum of 200 ng DNA and was run on Swift MaxPro Thermal Cycler (ESCO Healthcare, Singapore) with the following thermal profile [1]: activation at 95 °C for 10 min [2], amplification (38 cycles): denaturation at 95 °C for 1 min, annealing at 55 °C for 1 min, extension at 72 °C for 2 min and [3] final extension at 72 °C for 5 min. PCR products (BRAF, NRAS, c-KIT) were separated on 2% agarose gel. The band was excised and DNA purified using EZ-10 SPIN Column DNA Gel Extraction Kit (Bio Basic Inc., NY).

Samples bearing BRAF mutation by RFLP were evaluated by direct sequencing of the purified PCR product. BRAF wild type samples underwent NRAS exon 2, 3 sequencing and the double wild-type (BRAF, NRAS) samples were screened further for c-KIT exon 11, 13 mutations. The sequencing reaction was completed with BigDye Terminator v1.1 Cycle Sequencing Kit according to the manufacturer’s protocol on a 4-capillary automated sequencer (Applied Biosystems 3130 Genetic Analyzer) using the same primers for the PCR amplification reactions. Before analysis, purification of the sequencing reaction products was performed using BigDye XTerminatorTM Purification Kit. All kits, reagents and equipment used for sequencing were purchased from Life Technologies Corporation (Carlsbad, CA). Chromas Lite Version 2.1 software was applied to detect mutations compared to NCBI (National Center for Biotechnology Information) Nucleotide BLAST (Basic Local Alignment Search Tool) Human Database. The sensitivity of mutant-allele detection was determined as being 15%.

### Pyrosequencing and mutant allele fraction (MAF) determination

Paired primary and metastatic samples bearing different genotype (wild type/mutant in codon 600, 601 of BRAF and in codon 61 of NRAS gene) via Sanger sequencing were reanalysed with a higher sensitivity (> 2%) CE-IVD pyrosequencing technology. The Therascreen NRAS Pyro Kit and Therascreen BRAF Pyro Kit were used on the PyroMark Q24 System (Qiagen, Hilden, Germany) following instruction of the manufacturer Handbook. Sequencing primer for codon 601 of BRAF gene was also provided by the manufacturer. Primers for codon600/exon15 of BRAF and codon61/exon3 of NRAS were used. Five μl of genomic DNA was used in a total volume of 25 μl using 12.5 μl of 2x PyroMark PCR MasterMix, 2.5 μl 10xCoral-Load Concentrate, 1 ml PCR Primer of BRAF or NRAS and 4 μl of water Tied with the KIT. PCR was initiated by 15 min of 95 °C, followed by 42 cycles denaturation at 95 °C for 20s, annealing 53 °C for 30s and extension 72 °C for 20s, followed by final extension at 72 °C for 5 min. Ten μl of the PCR product was than subjected to the pyrosequencing reaction. Pyrogram outputs were analysed with the PyroMarkQ24 software (Qiagen) for the determination of the % of mutant allele vs wild type allele according to the relative peak heights of the corresponding nucleotides.

Importantly, the obtained PyroMark MAF value was corrected for the tumor/normal cell ratio in the given sample. Adjusted MAF value was calculated by multiplying PyroMark % by 100/x % tumor DNA. This adjusted MAF value was used throughout our experiments. Samples were also cathegorized into three artificial MAF categories: low (L) for less than 15% mutant allele, medium (M) for 15–40% mutant allele and high (H) for samples bearing more than 40% mutant allele [18].

### Statistical analysis

Statistical analysis was carried out using SPSS statistical package 20.0 (IBM Corporation, Chicago, IL, USA) software. A descriptive statistic was used to analyze the location specific distribution of driver mutations. For analysis of association between the amount of mutant alleles in primary and metastatic samples, paired t-probe (BRAF mutant samples) and nonparametric Wilcoxon sign rank test (NRAS mutant samples, because of the low case numbers) were performed as in case of the different locations of the metastases. Khi square and Fisher’s exact test were used for the analyze of correlation between three MAF categories (low, medium and high), based on MAF in primary and metastatic BRAF/NRAS mutant samples, and for the evaluation of the changes of MAF during tumor progression.

## Results

A total of 189 samples deriving from 50 patients with advanced melanoma were included in the analysis. Out of the 189 specimens 50 were primary cutaneous tumors (Table 1.) and 139 were corresponding haematogeneous metastases from 18 different visceral locations collected at autopsy (Table 2.). In our cohort study of primary-metastatic matched samples, 29 pairs had multiple distant metastases. A male dominance was observable and the mean age was 50 years. Four stage IB melanomas showed regression and almost 50% of primaries showed ulceration from this aggressive primary cohort (Table 1.). The most frequent metastatic organs were CNS, lung and liver, accounting for about half of the metastases, followed by adrenal gland, intestinal tract, distant skin, kidney and other rare sites (Table 2.). Regarding oncogenic drivers BRAF mutation was predominant (32/50, 64%) followed by NRAS (12/50, 24%), but no KIT mutant cases were found and triple wild type cases were in minority (6/50, 12%). BRAF mutations of primary melanomas were V600E (26/32, 81,25%) and V600K (5/32, 15,6%) and there was one case showing a rare codon 601 alteration (K601E). In NRAS mutant primaries Q61K (*n* = 5) and Q61R (n = 5) were equally frequent (5 cases each) and one case showed Q61L mutation and another carried codon 12 mutation (G12C). Survival of metastatic melanoma patients estimated by Kaplan-Meyer analysis was not affected by the driver status of the primary tumor (data not shown).

**Fig. 1:**
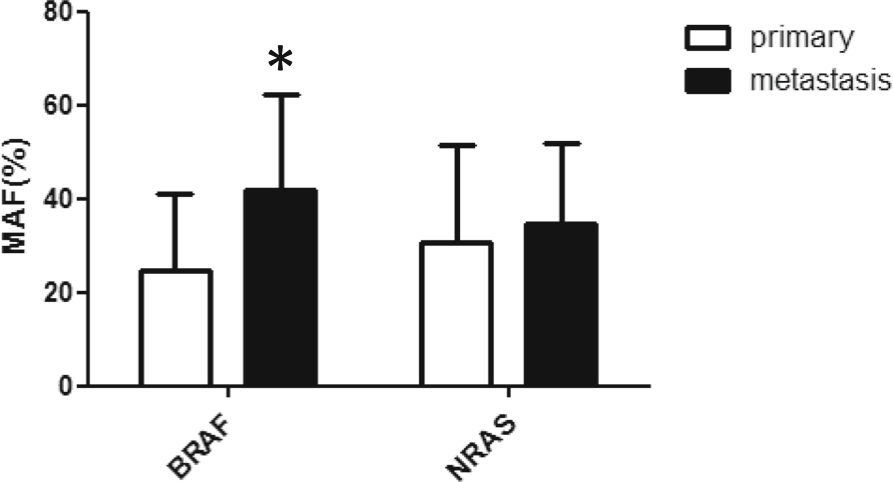
Mutant allele fraction (MAF) values of driver oncogenes, BRAF and NRAS in metastatic sites as compared with primary melanoma. Data represent mean+/−SD. * = *p* < 0.05 (Wilcoxon Signed Ranks Test)

**Fig. 2:**
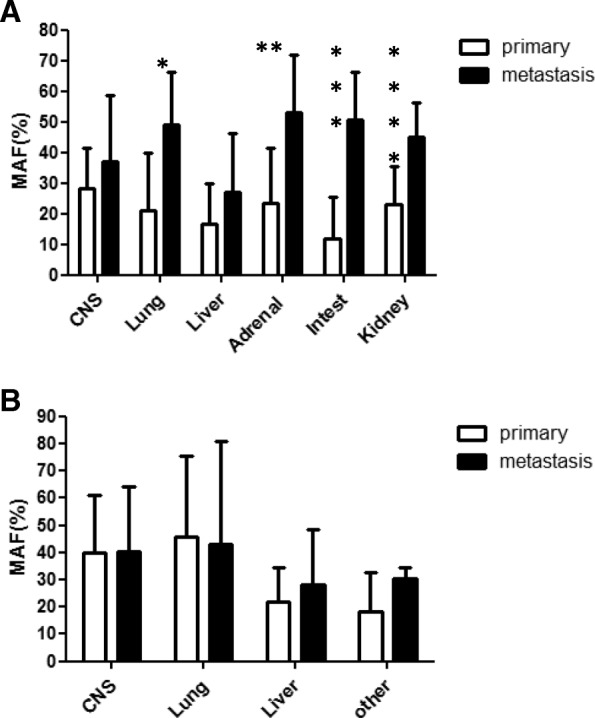
Mutant allele fraction (MAF) values of driver oncogenes (**a**: BRAF, **b**: NRAS) in major metastatic sites as compared with primary melanomas. Data represent mean+/−SD. The difference in BRAF mutant samples (**a**) is significant in case of: lung,*p = 0.001, adrenal gland,**p = 0.021, intestinal tract***p = 0.018, kidney****p = 0.043, based on Wilcoxon Signed Ranks Test. No significant differences were detected in NRAS mutant tumors (**b**)

BRAF-MAF was found to be in the range of 2.2–80.3%, while NRAS-MAF was in the range of 4.6–71.0%, indicating log range differences between various tumor samples. These large variations were not simply due to the variations in T/N ratio in the samples, since very low or very high T/N ratios were associated with extremely low MAF values and vice-versa (Table 1 and 2., Additional file 1: Table S1 and Additional file 2: Table S2) and MAF values were corrected for T/N ratio. Mean MAF was well below the expected 50% (heterozygosity) in primary tumors in case of both oncogenes: BRAF: 24.7+/− 16.3; NRAS: 30.7+/− 20.9. However, MAF of driver mutation was found to be significantly increased only in metastases of BRAF mutant melanomas (Fig. 1). When MAF values of the primary melanoma were compared to the most frequent metastatic sites, it was found that in case of BRAF mutant tumors the increase in MAF was due to a significant increase in lung-, adrenal-, intestinal- and kidney metastases (Fig. 2a), whereas no significant alterations were detected in metastatic sites of NRAS mutant tumors (Fig. 2b).

**Fig. 3:**
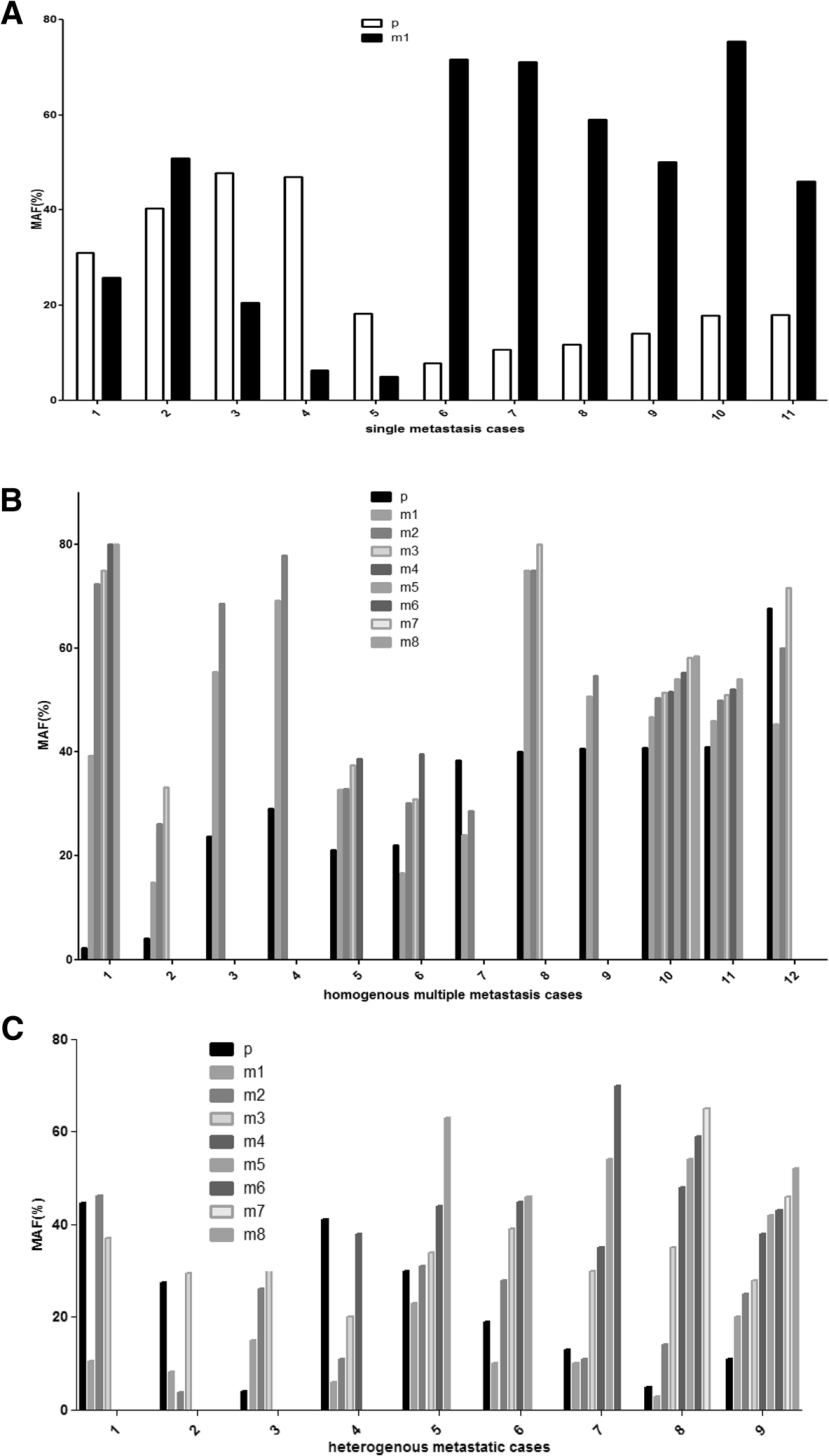
Case by case presentation of changes of BRAF mutant allele fraction (MAF) values of melanoma metastases compared with primary tumor. **a**. Single metastatic cases, p = primary tumor (white bar), m1 = metastatic tumor (black bar). **b**. Multiple homogeneous metastatic cases. **c**. Multiple heterogeneous metastatic cases. B/C p = primary (black bar), m1–8 = metastasis (shades of grey)

**Fig. 4:**
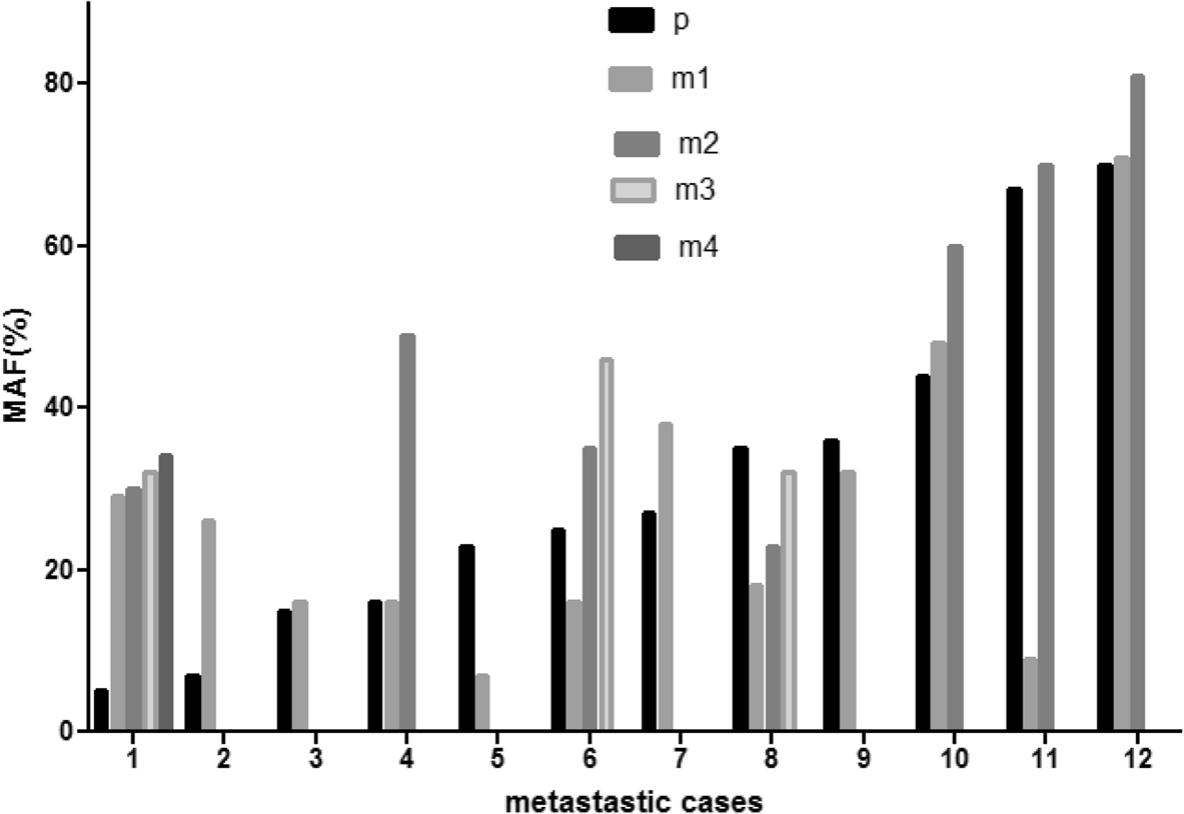
Case by case presentation of change of NRAS mutant allele fraction values of primary melanoma and metastases. p = primary tumor (black bar), m1–4 = individual metastasis (shades of grey)

Analysis of individual cases identified three different patterns. There were cases where metastases maintained the MAF profile of the primary tumor: BRAF mutant cases No1–2 on Fig. 3a, cases 5–12 on Fig. 3b. and NRAS-mutant cases 7–10 and − 12, (Fig. 4.). The other pattern was where a moderate shift of MAF was observable in the metastases: BRAF mutant cases 3,5,10,11 on Fig. 3a, cases 2–4 on Fig. 3b, NRAS mutant cases 2,3,5 on Fig. 4. The third pattern was where extreme MAF changes occurred in the metastases as compared to the primary tumor: BRAF mutant cases 4,6–9 on Fig. 3a., case No1 on Fig. 3b and cases 1,4 and 7–9 on Fig. 3c, NRAS mutants cases No4 and 11 on Fig. 4. (see also Additional file 1: Table S1 and Additional file 2: Table S2.) In this respect, no organ specificity could be identified. Finally, among the 129 metastases of 44 BRAF/NRAS mutant primaries, in case of two BRAF- and one NRAS-mutant tumors, in 4 out of the 129 metastases (3.1%) involving the spleen and liver the mutant driver oncogene allele was not detectable.

To better visualize these patterns, the individual samples were grouped into three MAF categories (Low, Medium and High). (Tables 3 and 4.) The three MAF variants were equally distributed in the primary melanomas irrespective of the oncogenic drivers. In Tables 3 and 4, one can not only see the primary to metastasis MAF alterations but also the fact that multiple metastases in case of both drivers could be either homogeneous (highly similar to each other: 23/32 in BRAF- Table 3. and 9/12 in NRAS-mutant cases, Table 4.) or heterogeneous (i.e. different): 9/32 in BRAF- (Table 3) and 3/12 in NRAS-mutant cases (Table 4.). Neither patterns showed statistically significant differences in case of the two drivers. Even more important is the finding that extreme MAF differences relative to primary (switch from high to low or low to high) in visceral metastases were relatively prevalent: 6/32 (18.75%) in BRAF-mutant homogenous cases, 4/32 in BRAF-mutant heterogenous cases (12.5%) (Table 3.) and 2/12 (16.7%) in NRAS-mutant cases. (Table 4). Furthermore, there were signs of positive selection in case of BRAF mutant melanomas during metastatic progression from the primary tumor, since medium to high (6/32), low to medium (3/32) and low to high (7/32) MAF shifts of metastases were more prevalent (16/32, 50.0%) as compared with the high to medium (3/32), medium to low (3/32) and high to low (1/32) MAF alterations (9/32, 28.1%). (Table 3.)

**Table 3:**
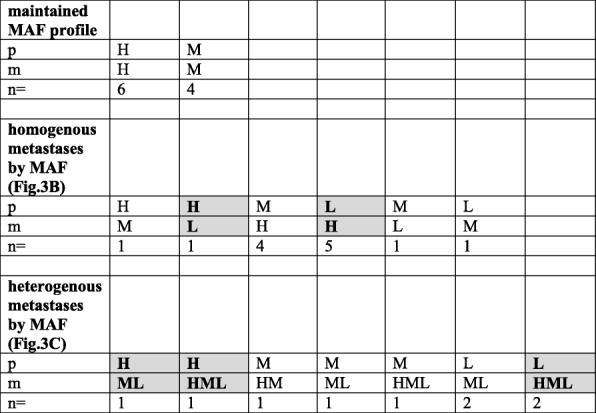
MAF patterns of BRAF mutant melanoma metastases compared to the primary

Patterns have been derived from Fig. 3. MAF classification: High was characterized by MAF > 40%, Medium referred to MAF of 15–40% while Low was defined as < 15% MAF. *p* primary tumor, *m* metastasis, *H* high, *M* medium, *L* low. *N* number of cases. Gray box = cases where extreme MAF changes (H to L or L to H) detected in individual metastasis

**Table 4:**
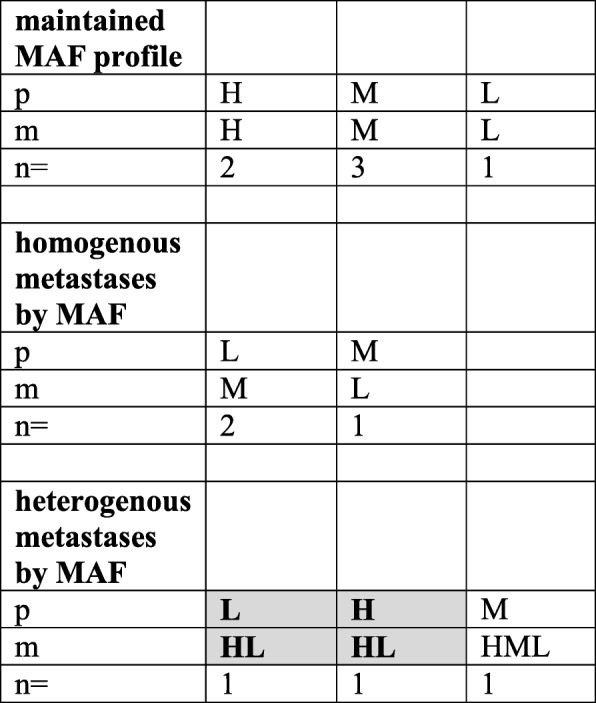
MAF patterns of NRAS mutant melanoma metastases compared to the primary

Patterns have been derived from Fig. 4. MAF classification: High was characterized by MAF > 40%, Medium referred to MAF of 15–40% while Low was defined as < 15% MAF. *p* primary tumor, *m* metastasis, *H* high, *M* medium, *L* low. *N* number of cases. Gray box = cases where extreme MAF changes (H to L or L to H) detected in individual metastasis

## Discussion

In line with previous findings, our present analysis of MAF profiles involving a large cohort of cutaneous malignant melanomas has confirmed that primary and visceral metastatic melanomas are both extremely heterogeneous for oncogenic drivers BRAF and NRAS, and this is neither due to technical issues nor to tumor-to-normal cell ratios [19]. Further, we demonstrated that BRAF-MAF was significantly increased in metastases suggesting a positive selection of mutant clones, which was not the case in NRAS mutant tumors. When MAF thresholds were applied (i.e. > 40% high, < 40 - > 15% medium, and low < 15%) this positive selection of BRAF mutant clones during metastatic progression became more evident, since no difference was found in the incidence of the three MAF variants in the primaries, while in case of metastases the high MAF variant became predominant (15/32, 46.8%) and low MAF dropped to 2/32, 6.25%. MAF values significantly higher than 50% or lower than 15% were frequent both in primary as well as metastatic melanoma. The extremely high MAF may suggest amplification of the mutant gene or LOH of the wild type one. On the other hand, the extremely low MAF values may also suggest LOH of the mutant or amplification of the wild type gene. An ongoing study on CNV characterization will answer these questions.

Our study is the first to demonstrate that BRAF mutant melanoma clones are significantly enhanced in organs such as the lung, adrenal gland, intestine or kidney but not in the CNS or liver, which suggests that organ-specific genetic mechanisms are operational during the metastatic progression of melanomas. Our data differ from those reported recently, according to which - based on the mutational status in the primary tumor - BRAF- and NRAS-mutant tumors more often develop metastasis to the CNS and liver and NRAS mutant tumors have been associated with lung metastasis [20]. It would be interesting to see if MAF of the driver oncogenes in the primary tumor has any prognostic role in this respect.

In our study, we had the opportunity to analyse multiple visceral metastases of the same BRAF or NRAS mutant primary tumors. It was found that in a significant proportion of multiple metastatic cases an extreme inter-metastasis heterogeneity for MAF existed (BRAF: 28.2%, NRAS: 25%) although the majority of metastatic cases were homogeneous.

Our further observations support the experience that BRAF and NRAS mutant melanomas behave differently during metastatic progression. In half of the NRAS mutant cases, metastases maintained the MAF profile of the primary tumor, while this was not so common in the BRAF mutant cases (31.3%). Furthermore, in a significant proportion of BRAF mutant cases metastases switched from low to high or high to low MAF and such an extreme shift also occurred individually in heterogeneous multiple metastatic BRAF mutant cases, unlike in NRAS mutant melanomas.

Our data are not only important from the theoretical point of metastatic progression and clonal dynamics [21, 22] but also from the viewpoint of clinical decision-making. In case of melanoma, targeted therapy is based on the determination of the driver mutational status. BRAF inhibitors are clinically active in BRAF-mutant melanomas, but only in a fraction of patients and the effect is transient [23, 24]. One of the cause of this ineffectiveness could well be the extreme heterogeneity of the MAF values of BRAF, which in our case was in the range of 2.2–80.3%. Furthermore, considering cases with multiple metastases, the inter-metastatic heterogeneity of MAF values is suggestive of the presence of such secondary tumors, which fail to respond optimally to targeted therapy. This metastatic heterogeneity however, can also be present at a lower level in case of NRAS mutant melanoma. Currently, in a significant proportion of cases the oncogenic driver mutation status is determined from the primary tumor. In our study, loss of the driver oncogene in the metastatic tissues was an extremely rare finding (3.1%) at our technical threshold of 2%, which might support this rationale. However, the MAF proportion is rather heterogeneous in primary tumors and metastases and is independent of the tumor/normal ratios, which cannot predict the situation in metastatic tissues. It is our opinion that among the reasons for the relatively low efficiency of targeted therapies for malignant melanoma are clonal heterogeneity and low MAF rate of driver oncogenes in the metastases. Regarding melanomas, it would be important to determine the predictive value of MAF in the effectiveness of targeted therapies within a larger retrospective study, since our cohort study comprised only 3 cases treated with BRAF inhibitor.

## Conclusions

The data reported in this study suggest that in visceral metastases of malignant melanoma BRAF- or NRAS-mutant allele fractions are rather heterogeneous and are quite difficult to predict based on data from the primary tumor. These findings may bear significance in the clinical use of targeted therapies.

## Additional files

Additional file 1: **Table S1.** Tumor to normal ratios and adjusted MAF values of BRAF mutant samples. T/N ratio, measured mutant BRAF MAF values and adjusted/calculated MAF values of individual cases are presented. (DOCX 17 kb)

Additional file 2: **Table S2.** Tumor to normal ratios and adjusted MAF values of NRAS mutant samples. T/N ratio, measured mutant NRAS MAF values and adjusted/calculated MAF values of individual cases are presented. (DOCX 14 kb)

## Data Availability

The datasets used in this publication are available from the corresponding author upon reasonable request.

## Abbreviations

CNS: Central nervous system
LOH: loss of heterozygosity
MAF: mutant allele fraction
T/N: Tumor to normal cell ratio
