# Correction to: Dynamic and unpredictable changes in mutant allele fractions of BRAF and NRAS during visceral progression of cutaneous malignant melanoma

**DOI:** 10.1186/s12885-019-6048-8

**Published:** 2019-08-29

**Authors:** V. Doma, S. Kárpáti, E. Rásó, T. Barbai, J. Tímár

**Affiliations:** 1 2nd Department of Pathology, Semmelweis University, 93. Üllői, Budapest, H-1091 Hungary; 2 Department of Dermatology, Semmelweis University, Budapest, Hungary

**Correction to: BMC Cancer (2019) 19:786**

**https://doi.org/10.1186/s12885-019-5990-9**

Following publication of the original article [1], the authors reported the family name of the second author was incorrectly published.

*Incorrect:* S. Kárpáthy

*Correct:* S. Kárpáti

The original article [1] has been corrected.
