# Supplementary material for: Dynamic and unpredictable changes in mutant allele fractions of BRAF and NRAS during visceral progression of cutaneous malignant melanoma
Source: BMC Cancer. 2019 Aug 7;19:786. doi: 10.1186/s12885-019-5990-9 (PMC6686548; doi:10.1186/s12885-019-5990-9)
Supplement: Supplementary file 1 — Table S1. Tumor to normal ratios and adjusted MAF values of BRAF mutant samples. T/N ratio, measured mutant BRAF MAF values and adjusted/calculated MAF values of individual cases are presented. (DOCX 17 kb) [file 12885_2019_5990_MOESM1_ESM.docx]

***Additional file 1 Table S 1. Tumor to normal ratios and adjusted MAF values of BRAF mutant samples***

| caseBRAF | sample | T/N% | aMAF% |
| --- | --- | --- | --- |
|  |  |  |  |
| 1 | PR | 83 | 22.1 |
|  | MLU | 92 | 30.9 |
|  | MA | 93 | 30.2 |
|  | MOV | 91 | 16.7 |
|  | MC | 95 | 39.6 |
| 2 | PR | 93 | 31.0 |
|  | MC | 91 | 25.8 |
| 3 | PR | 94 | 38.4 |
|  | MBR | 84 | 26.2 |
|  | MOV | 85 | 34.9 |
| 4 | PR | 93 | 38.4 |
|  | MBR | 91 | 28.6 |
|  | MBR | 84 | 24.0 |
| 5 | PR | 95 | 41.0 |
|  | MBR | 53 | 10.9 |
|  | MBR | 34 | 5.8 |
|  | MOV | 52 | 20.4 |
|  | MC | 91 | 37.5 |
| 6 | PR | 83 | 44.6 |
|  | MBR | 94 | 37.1 |
|  | MLU | 92 | 46.2 |
|  | MOV | 22 | 10.6 |
| 7 | PR | 93 | 21.2 |
|  | MBR | 90 | 35.7 |
|  | MBR | 91 | 32.8 |
|  | MLU | 93 | 37.5 |
|  | MA | 91 | 32.7 |
|  | MK | 24 | 38.6 |
| 8 | PR | 92 | 19.4 |
|  | MBR | 91 | 48.5 |
|  | MBR | 94 | 10.5 |
|  | MBR | 62 | 26.2 |
|  | MLU | 91 | 44.6 |
|  | MOV | 93 | 28.5 |
|  | MOV | 92 | 46.1 |
|  | MC | 91 | 39.0 |
| 9 | PR | 89 | 30.0 |
|  | MBR | 88 | 63.0 |
|  | MLI | 49 | 23.4 |
|  | MA | 52 | 34.1 |
|  | MK | 47 | 31.3 |
|  | MC | 63 | 44.3 |
| 10 | PR | 72 | 27.4 |
|  | MLU | 84 | 29.5 |
|  | MLI | 88 | 8.3 |
|  | MOV | 59 | 3.8 |
| caseBRAF | sample | T/N% | aMAF% |
|  |  |  |  |
| 11 | PR | 87 | 67.6 |
|  | MLU | 78 | 45.4 |
|  | MA | 91 | 60.0 |
|  | MOV | 92 | 71.6 |
| 12 | PR | 83 | 12.9 |
|  | MLI | 79 | 34.8 |
|  | MA | 93 | 70.0 |
|  | MK | 89 | 54.5 |
|  | MOV | 12 | 2.3 |
|  | MOV | 53 | 30.3 |
|  | MOV | 92 | 9.6 |
|  | MOV | 48 | 11.6 |
| 13 | PR | 51 | 4.0 |
|  | MLU | 84 | 26.2 |
|  | MLU | 72 | 33.1 |
|  | MLI | 47 | 14.9 |
| 14 | PR | 28 | 3.5 |
|  | MLU | 67 | 36.3 |
|  | MLI | 48 | 22.6 |
|  | MLI | 68 | 30.3 |
|  | MOV | 55 | 26.0 |
| 15 | PR | 86 | 4.7 |
|  | MBR | 11 | 2.5 |
|  | MLU | 86 | 54.1 |
|  | MLI | 34 | 14.0 |
|  | MOV | 86 | 64.7 |
|  | MOV | 92 | 59.3 |
|  | MOV | 13 | 2.2 |
|  | MOV | 77 | 48.4 |
|  | MC | 56 | 35.4 |
| 16 | PR | 76 | 11 |
|  | MBR | 89 | 24.3 |
|  | MLU | 78 | 37.8 |
|  | MLI | 83 | 28.0 |
|  | MA | 87 | 53.6 |
|  | MK | 78 | 43.5 |
|  | MOV | 84 | 18.8 |
|  | MOV | 86 | 52.9 |
|  | MOV | 67 | 41.9 |
|  | MOV | 79 | 37.8 |
|  | MI | 92 | 57.6 |
| 17 | PR | 83 | 4.3 |
|  | MLU | 91 | 75.4 |
|  | MA | 88 | 75.4 |
|  | MOV | 92 | 40.1 |
|  | MC | 93 | 79.9 |
|  |  |  |  |
|  |  |  |  |
| caseBRAF | sample | T/N% | aMAF% |
| 18 | PR | 18 | 2.2 |
|  | MLU | 87 | 80.0 |
|  | MA | 92 | 72.4 |
|  | MI | 47 | 39.3 |
|  | MOV | 94 | 80.3 |
|  | MOV | 86 | 74.9 |
| 19 | PR | 88 | 10.6 |
|  | MLU | 91 | 71.1 |
| 20 | PR | 68 | 7.8 |
|  | MBR | 89 | 71.6 |
| 21 | PR | 76 | 11.7 |
|  | MI | 87 | 59.0 |
| 22 | PR | 52 | 14.0 |
|  | MBR | 92 | 50.1 |
| 23 | PR | 74 | 17.8 |
|  | MBR | 93 | 75.4 |
| 24 | PR | 87 | 40.3 |
|  | MBR | 90 | 50.9 |
| 25 | PR | 88 | 23.7 |
|  | MBR | 84 | 68.6 |
|  | MBR | 82 | 55.4 |
| 26 | PR | 93 | 29.1 |
|  | MLU | 87 | 77.9 |
|  | MLI | 91 | 69.1 |
| 27 | PR | 86 | 40.8 |
|  | MBR | 89 | 54.1 |
|  | MLU | 94 | 55.3 |
|  | MLI | 77 | 50.4 |
|  | MA | 89 | 46.7 |
|  | MK | 92 | 58.1 |
|  | MI | 85 | 51.4 |
|  | MOV | 91 | 51.6 |
|  | MOV | 95 | 58.5 |
| 28 | PR | 48 | 18 |
|  | MBR | 87 | 46.2 |
| 29 | PR | 91 | 40.7 |
|  | MBR | 93 | 50.7 |
|  | MLU | 89 | 54.6 |
| 30 | PR | 86 | 47.8 |
|  | MBR | 78 | 20.6 |
| 31 | PR | 84 | 18.2 |
|  | MLI | 88 | 5.1 |
| 32 | PR | 90 | 46.9 |
|  | MBR | 86 | 6.3 |

T/N= tumor to normal ratio expressed in %, aMAF= measured MAF values adjusted to the T/N ratio as expressed in%, PR= primary tumor, MBR= brain metastasis, MLU= lung metastasis, MLI= liver metastasis, MA= adrenal gland metastasis, MK= kidney metastasis, MI= intestinal metastasis, MC= distant cutaneous metastasis, MOV= other visceral metastasis
