# Supplementary material for: Dynamic and unpredictable changes in mutant allele fractions of BRAF and NRAS during visceral progression of cutaneous malignant melanoma
Source: BMC Cancer. 2019 Aug 7;19:786. doi: 10.1186/s12885-019-5990-9 (PMC6686548; doi:10.1186/s12885-019-5990-9)
Supplement: Supplementary file 2 — Table S2. Tumor to normal ratios and adjusted MAF values of NRAS mutant samples. T/N ratio, measured mutant NRAS MAF values and adjusted/calculated MAF values of individual cases are presented. (DOCX 14 kb) [file 12885_2019_5990_MOESM2_ESM.docx]

***Additional file 2: Table S 2. Tumor to normal ratios and adjusted MAF values of NRAS mutant samples***

| case | sample | T/N% | aMAF% |
| --- | --- | --- | --- |
| 1 | PR | 87 | 69.6 |
|  | MBR | 92 | 71.0 |
|  | MBR | 86 | 81.3 |
| 2 | PR | 38 | 35.4 |
|  | MBR | 87 | 22.8 |
|  | MLI | 92 | 38 |
|  | MK | 88 | 32.5 |
|  | MOV | 86 | 31.9 |
|  | MC | 47 | 18.6 |
| 3 | PR | 85 | 14.7 |
|  | MBR | 53 | 16.3 |
| 4 | PR | 87 | 26.6 |
|  | MBR | 93 | 38.4 |
| 5 | PR | 83 | 35.8 |
|  | MBR | 91 | 31.9 |
| 6 | PR | 86 | 24.8 |
|  | MLU | 89 | 16.0 |
|  | MLI | 91 | 46.0 |
|  | MOV | 84 | 35.3 |
| 7 | PR | 86 | 43.5 |
|  | MBR | 91 | 60.0 |
|  | MBR | 87 | 47.6 |
| 8 | PR | 68 | 4.6 |
|  | MLI | 86 | 29.3 |
|  | MA | 91 | 34.4 |
|  | MOV | 84 | 32.3 |
|  | MI | 81 | 30.5 |
| 9 | PR | 91 | 7.7 |
|  | MOV | 76 | 26.0 |
| 10 | PR | 74 | 15.8 |
|  | MBR | 88 | 49.4 |
|  | MBR | 29 | 16.6 |
| 11 | PR | 92 | 66.6 |
|  | MBR | 67 | 8.75 |
|  | MLU | 86 | 69.8 |
| 12 | PR | 88 | 23.0 |
|  | MLI | 92 | 7.5 |

T/N= tumor to normal ratio expressed in %, aMAF= measured MAF values adjusted to the T/N ratio as expressed in%, PR= primary tumor, MBR= brain metastasis, MLU= lung metastasis, MLI= liver metastasis, MA= adrenal gland metastasis, MK= kidney metastasis, MC= distant cutaneous metastasis, MOV= other visceral metastasis
